# Pasireotide—a novel somatostatin receptor ligand after 20 years of use

**DOI:** 10.1007/s11154-022-09710-3

**Published:** 2022-01-24

**Authors:** Marek Bolanowski, Marcin Kałużny, Przemysław Witek, Aleksandra Jawiarczyk-Przybyłowska

**Affiliations:** 1 Department of Endocrinology, Diabetes and Isotope Therapy, Wrocław Medical University, Wrocław, Poland; 2 Department of Internal Medicine, Endocrinology and Diabetes, Mazovian Bródno Hospital, Medical University of Warsaw, Warsaw, Poland

**Keywords:** Pasireotide, Acromegaly, Cushing’s disease, Adverse effects, Hyperglycemia

## Abstract

Pasireotide, a novel multireceptor-targeted somatostatin receptor ligand (SRL) is characterized by a higher affinity to somatostatin receptor type 5 than type 2, unlike first-generation SRLs. Because of the broader binding profile, pasireotide has been suggested to have a greater clinical efficacy in acromegaly than first-generation SRLs and to be efficacious in Cushing’s disease. The consequence of this binding profile is the increased blood glucose level in some patients. This results from the inhibition of both insulin secretion and the incretin effect and only a modest suppression of glucagon. A monthly intramuscular formulation of long-acting release pasireotide has been approved for both acromegaly and Cushing’s disease treatment. This review presents data on the efficacy and safety of pasireotide treatment mostly in patients with acromegaly and Cushing’s disease. Moreover, other possible therapeutic applications of pasireotide are mentioned.

## Somatostatin and its receptors

Somatostatin (SST) plays an important role in the regulation of the endocrine system and in the functioning of the gastrointestinal tract. In different organs somatostatin acts as a neurohormone, a neurotransmitter, or a local factor via autocrine or paracrine signaling. Endogenous SST consists of 14 or 28 amino acids, has a short half-life of 1–3 min, and is a physiological inhibitor of growth hormone (GH) secretion. SST counteracts growth hormone releasing hormone (GHRH) effects on GH secretion by pituitary somatotroph cells. Moreover, SST can decrease the pituitary secretion of thyroid stimulating hormone (TSH) and prolactin. In the gastrointestinal system SST inhibits the secretion of cholecystokinin, gastric inhibitory peptide, gastrin, motilin, neurotensin, secretin, glucagon, insulin, and pancreatic polypeptide. It also inhibits the exocrine function of the gastrointestinal mucosa, salivary glands, and liver; inhibits and modulates gastrointestinal absorption and motility; and decreases portal pressure [1–3].

The antisecretory effects of SST occur through adenylyl cyclase inhibition, calcium channel blockade, and potassium channel stimulation [4, 5]. The clinical effects of SST are mediated by its specific receptors. There are 5 subtypes of somatostatin receptors (SSTR1–5) that belong to the G protein-coupled receptor superfamily. Out of those, only SSTR2 and SSTR5 play a significant clinical role. Endogenous SST has a comparable binding affinity to all of its receptors. Somatotropinomas express SST receptors, especially SSTR2 and SSTR5 [2, 6, 7]. The development of somatostatin analogs (SAs), also known as somatostatin receptor ligands (SRLs), has brought the opportunity to control hormonal hypersecretion via SST receptors. Various SRLs display different levels of affinity for the individual types of SST receptors, with first-generation SRLs (octreotide - OCT and lanreotide - LAN) binding mostly to SSTR2 and SSTR5 while exhibiting a moderate affinity to SSTR3 and a low affinity to SSTR1 and SSTR4. The novel SRL pasireotide (PAS) binds with a high affinity to all SST receptor subtypes, with the exception of SSTR4 [8–10].

## First-generation somatostatin receptor ligands

SRL synthesis was an important milestone in medical treatment of acromegaly. At the beginning, OCT was shown to suppress GH secretion by somatotropes [11]. The first generation short-acting SRLs OCT and LAN were the first ones developed [12, 13]. Both show a high affinity to SSTR2 and SSTR5 and a weak affinity to SSTR3. These agents constitute first-line treatments for a majority of acromegaly patients [14, 15]. However, long-acting SRL formulations are much more useful in clinical practice. They are lanreotide prolonged release, injected once every two weeks, and octreotide long-acting release (OCT-LAR) and lanreotide autogel (LAN-ATG), injected monthly [16, 17]. The latter two formulations are very convenient for the patients and represent the medical therapy of choice for a majority of patients with acromegaly. They are considered as an adjuvant therapy (following failed neurosurgery), the primary medical therapy (when surgery is not suitable), or a neoadjuvant therapy (prior to surgery) in mono- or combination therapy [14, 15]. Although the first-generation SRLs control hormonal hypersecretion in about half of the acromegaly patients, they promote tumor shrinkage and improve cardiovascular and metabolic comorbidities [18–20]. Recently various new OCT forms have been applied. An oral OCT has demonstrated its efficacy and safety in acromegaly patients controlled with an injectable SRL formulation. Moreover, an oral route of administration gives a treatment option to patients intolerant of or reluctant to take monthly injections [21–23]. Another OCT form, subcutaneous implants were reported to sustain a stable OCT concentration for 6 months and normalize GH and insulin-like growth factor 1 (IGF-1) levels in 86% and 84% of patients, respectively. The safety profile was similar to that of OCT-LAR therapy [24].

## Second-generation somatostatin receptor ligand – pasireotide

**Fig. 1:**
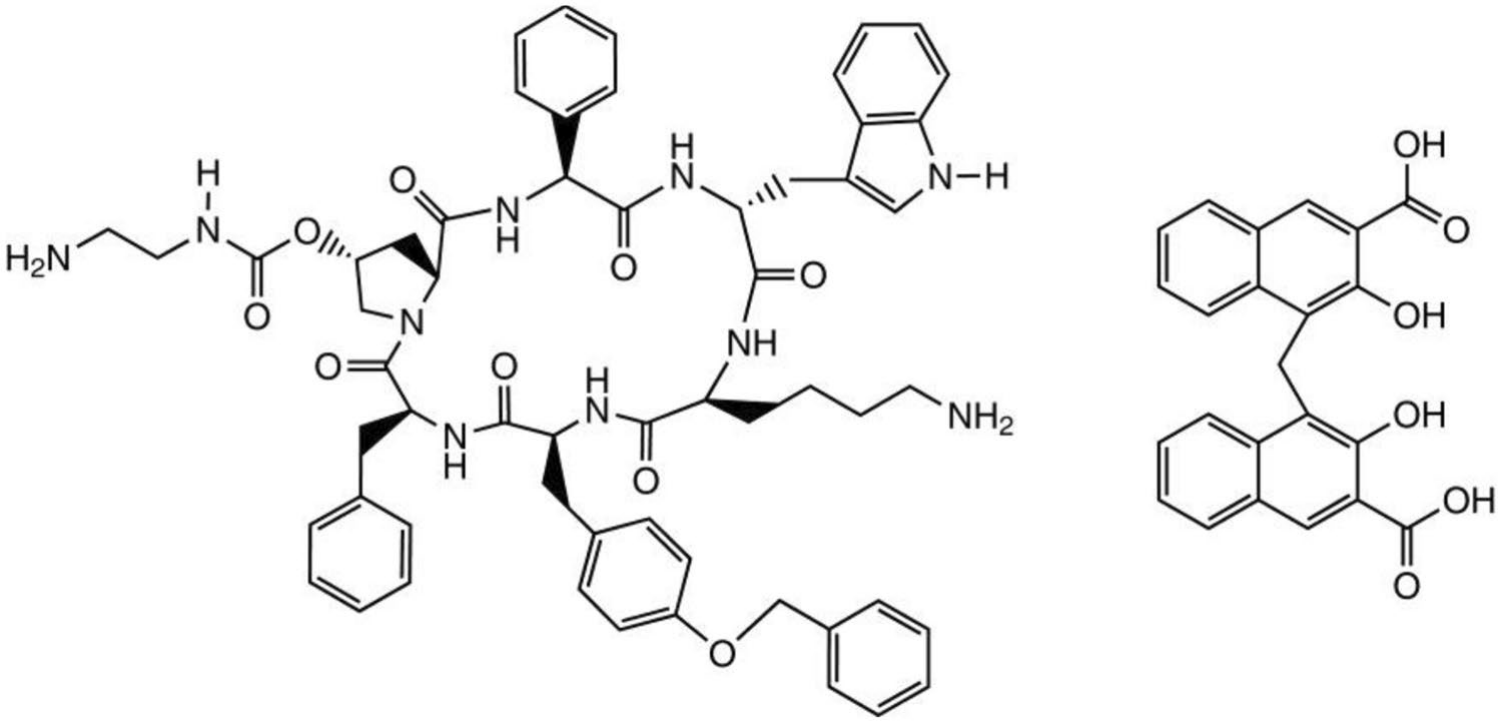
The structural formula of pasireotide (from: Signifor LAR Highlights of, prescribing information, revised April 2019)

**Fig. 2:**
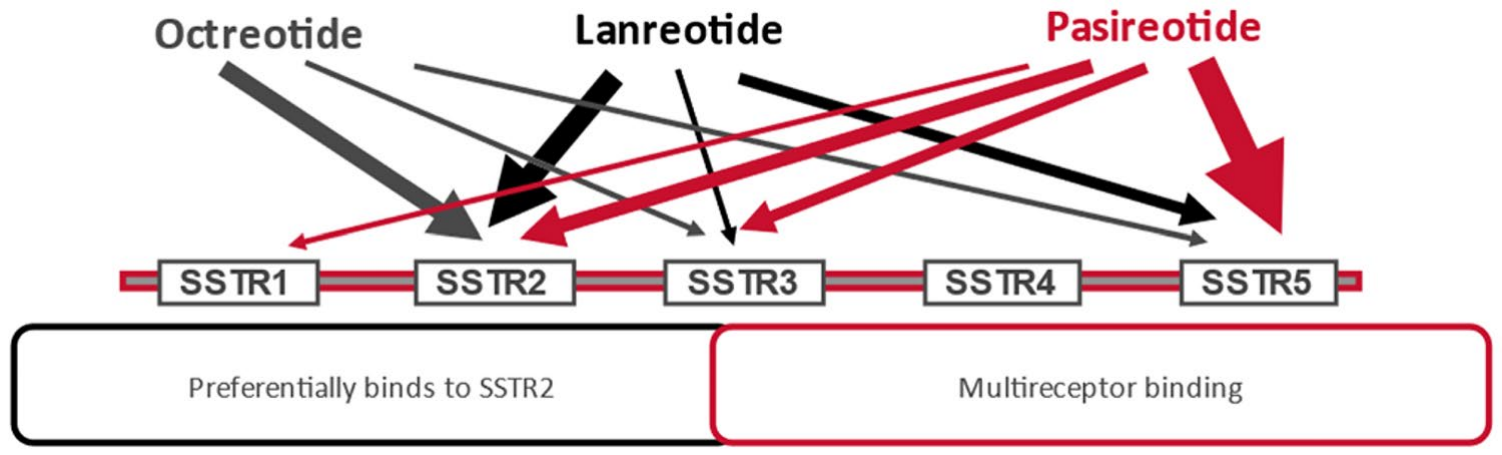
Comparison of different somatostatin receptor ligands binding affinities to subtypes of somatostatin receptors (materials from Recordati, modified) [5, 8, 9]

Pasireotide (Fig. 1) is a novel multireceptor-targeted SRL characterized by a higher affinity to SSTR5 than to SSTR2, unlike first-generation SRLs. In comparison with OCT, PAS exhibits a 158-fold higher affinity to SSTR5 but a sevenfold lower affinity to SSTR2 (Fig. 2). PAS shows a half-life of 7–11 h following a single subcutaneous administration [8, 25]. Because of the broader binding profile, PAS-LAR has been suggested to have a greater clinical efficacy in acromegaly than first-generation SRLs and to be efficacious in Cushing’s disease [26, 27]. A monthly intramuscular formulation has been approved for both acromegaly and, more recently, Cushing’s disease. PAS targets four of the five SST receptor subtypes, with the highest affinity for SSTR5, followed by SSTR2 [8]. The binding affinity to SSTR5 is several times higher for PAS than it is for either OCT or LAN, which explains the increased efficacy of PAS in patients with acromegaly or Cushing’s disease, by reduction of GH and adrenocorticotropic hormone (ACTH) secretion, respectively [26, 27]. Another point of this binding profile is the increased blood glucose in some patients [28]. This results from the inhibition of both insulin secretion and the incretin effect and only a modest suppression of glucagon, all of which are reversible upon discontinuation of PAS [29, 30]. In a study on healthy volunteers, subcutaneous PAS administration at doses of 600, 900, or 1,200 µg twice daily for 7 days resulted in a significant decrease in insulin secretion. The suppression of glucagon was less pronounced. No changes in hepatic or peripheral insulin sensitivity were shown during a hyperinsulinemic-euglycemic clamp. Moreover, an increase in the glucose area under the curve (AUC) and a decrease in glucagon-like peptide 1 (GLP-1) AUC and glucose-dependent insulinotropic polypeptide (GIP) levels were found during an oral glucose tolerance test (OGTT) [31]. The hyperglycemic effect of PAS can be explained by the drug’s binding-affinity profile. Glucagon-producing alpha cells predominantly express SSTR2, whereas insulin-producing beta cells mainly express SSTR2 and SSTR5. By its high-affinity binding to SSTR5, PAS potently suppresses insulin secretion, whereas the drug’s inhibitory effect on glucagon secretion is only modest, PAS does not influence insulin resistance [29, 31, 32]. Before starting PAS therapy, patients should undergo an assessment of glucose metabolism and, in diabetic patients, anti-diabetic treatment should be initiated or optimized [14, 28].

## Pasireotide in the treatment of acromegaly

The current therapeutic guidelines consider the effectiveness of PAS-LAR in the treatment of acromegaly and recommend the use of this drug as second-line treatment [15, 26, 33, 34]. The populations likely to benefit most from PAS-LAR treatment are young people who showed tumor growth during first-generation SRL treatment and patients with severe headaches who failed to respond to or were intolerant of previous medical treatments. Another important group of candidates for an attempt at PAS-LAR monotherapy are non-diabetic patients receiving a combination therapy of a first-generation SRL and low-dose pegvisomant (PEGV < 80 mg/week). PAS-LAR may be also used in combination with PEGV if other therapeutic options are inadequate [15, 35]. Studies show that PAS may be a better therapeutic option in rare types of acromegaly associated with large pituitary tumors, e.g. X-linked acrogigantism (X-LAG) or aryl hydrocarbon receptor-interacting protein (AIP) mutation-positive acromegaly [36]. High efficacy of PAS in normalizing GH and IGF-1 levels has been demonstrated in both preclinical and phase II clinical studies. Van der Hoek et al. used single subcutaneous doses of PAS and OCT in 12 patients with active acromegaly to assess the extent of GH and IGF-1 suppression. Their study showed that single-dose subcutaneous PAS at 100 and 250 μg induced a dose-dependent GH and IGF-1 suppression. The effects of PAS and OCT were comparable in eight patients; however, in three patients the suppressive effect on GH secretion was considerably greater with subcutaneous PAS [37]. Another study, a phase II randomized crossover study involved 60 patients with active acromegaly who either underwent prior neurosurgery or radiotherapy or had no previous treatment. Initially, all patients received 100 μg OCT subcutaneously three times a day for 28 days and then received PAS at 200, 400, and 600 μg subcutaneously twice daily in random order for 28 days. A biochemical response was defined if mean GH measured 1, 1.5 and 2 h after administration of study drug was no more than ≤ 2.5 μg/L and age- and sex-adjusted normalization of IGF-1 levels. After four weeks of OCT administration, a biochemical response was achieved in 9% of patients. In the case of PAS administered at 200–600 μg, biochemical control was shown in 19% at week 4 and in 27% at month 3 of treatment. Although the high-dose group showed higher rates of both complete and partial responses to PAS treatment, no clear correlation was observed between the dose and the achieved biochemical response. Thirty-nine percent of patients achieved a more than 20% reduction in tumor volume; however, the authors were unable to indisputably distinguish the possible earlier OCT effect on tumor size reduction [38].

### Patients with no previous medical treatment

A phase III trial (CS2305) was conducted in patients naive to medical treatment, either after an unsuccessful surgery or with newly diagnosed acromegaly. These patients received 40 mg PAS-LAR every 28 days (*n* = 176) or 20 mg OCT-LAR every 28 days (*n* = 182) for 12 months. A dose increase to PAS-LAR 60 mg or OCT-LAR 30 mg was permitted but no mandatory at month 3 and 7 based on biochemical response (mean GH ≥ 2.5 μg/L and/or IGF-1 above the upper limit of normal [ULN]). The rates of biochemical response were significantly higher with PAS-LAR than with OCT-LAR (31.3% vs. 19.2%, respectively; *p* = 0.007). The efficacy of PAS in normalizing IGF-1 levels was considerably higher (38.6% vs. 23.6%, *p* = 0.002); however, both drugs showed comparable efficacy in lowering GH levels below 2.5 μg/L (48.3% vs. 51.6%). PAS-LAR and OCT-LAR therapy helped achieve normal IGF-1 levels in 50.7% and 26.9% of patients after surgery, respectively, and in 30.5% vs. 21.2% of treatment-naive patients, respectively. Treatment efficacy might have been even higher in both groups, considering the fact that 31% of PAS-LAR patients and 22.2% of OCT-LAR patients had not had their doses increased despite of a lack of disease control with the initial dosage [26]. The subsequent extension study evaluated the population of patients who had not achieved disease control with PAS or OCT treatment. The extension study involved 119 patients, with 81 patients switched to PAS-LAR treatment and 38 patients switched to OCT-LAR treatment. Following the switch to PAS, 14 patients (17.3%) achieved complete disease control (GH < 2.5 μg/L and normalized IGF-1 levels), 36 patients (44.4%) showed GH level reduction to < 2.5 μg/L, and 22 patients (27.2%) achieved IGF-1 level normalization. Out of the 38 patients in whom the initial PAS treatment proved ineffective, none achieved complete disease control following the switch to OCT, nine patients (23.7%) showed GH level reduction to < 2.5 μg/L and two patients (5.3%) achieved normal IGF-1 levels [39]. Both studies demonstrated a superior efficacy of PAS-LAR in comparison with OCT-LAR in normalizing IGF-1 levels, with comparable effects of both drugs on GH level reduction. This is consistent with *in vitro* study results, which showed both drugs to be comparably effective in reducing GH secretion [40, 41]. Moreover, 80% and 77% of patients treated with PAS-LAR and OCT-LAR, respectively, achieved significant tumor mass reduction. The mean achieved tumor volume reduction was 40% in both groups [26].

### Patients ineffectively treated with first-generation SRLs

The PAOLA double-blind trial (C2402) compared the efficacy of PAS-LAR (at 40 or 60 mg) to that of an active control. The control group were patients who continued to receive a first-generation SRL (OCT or LAN) despite a lack of disease control after 6 months of SRL treatment. Inadequate disease control was defined as the mean GH value of > 2.5 μg/L from a five-point profile of 2 h and IGF-1 levels of > 1.3 × ULN adjusted for sex and age. At month 6 of 40 mg or 60 mg PAS-LAR therapy, biochemical control was achieved in both dosage groups, with 15% and 20% patients, respectively, achieving not only complete biochemical control, but also meeting the GH and IGF-1 level criteria after six months of treatment, in comparison with 0% of patients from the control group prior to treatment. Tumor reduction or no change in tumor size from baseline to month 6 of treatment (assessed with magnetic resonance imaging [MRI] of the pituitary gland) was achieved in 81% and 70% of patients treated with PAS-LAR at 40 mg and 60 mg, respectively. A tumor volume reduction by 25% or more was achieved by week 24 in a greater proportion of patients from the PAS group than that from the first-generation SRL group (in 18% of the 40 mg subgroup and 11% of the 60 mg subgroup of PAS-LAR treatment vs. 1.5% of the first-generation SRL group) [42]. The extension study evaluated 173 patients receiving PAS-LAR, and the efficacy and safety follow-up was continued for a mean of 304 weeks (5.8 years) in the 111 patients who had been receiving PAS from the beginning (the initial study and the extension study) or 268 weeks (5.2 years) for the 62 patients who were switched to PAS treatment in the extension study. Based on the disease control criterion of achieving GH levels of < 1 μg/L and IGF-1 levels within normal limits for age and sex, acromegaly control was achieved in 37% patients, 65.5% of whom achieved it after at least six months of PAS-LAR treatment. A switch from first-generation SRLs to PAS-LAR helped achieve disease control in 22% of patients, and PAS-LAR dose increase from 40 to 60 mg produced this effect in 28% of patients. Moreover, all study groups showed improvement in important acromegaly-related symptoms (headaches, fatigue, diaphoresis, paresthesia, osteodynia, and arthralgia) [43]. Another phase III study confirmed the efficacy of PAS-LAR in patients inadequately controlled with maximum doses of first-generation SRL (OCT or LAN for ≥ 3 months). The study included 123 patients, 113 of whom had completed the main study and 88 continued their treatment as part of an extension study. A total of 18 patients (14.6%) achieved mean GH (mGH) levels of < 1 μg/L and IGF-1 levels < ULN by week 36; biochemical control was achieved in 42.9% of patients with baseline mGH of 1.0–2.5 μg/L and in 6.4% of patients with baseline mGH of > 2.5 μg/L. The groups of patients previously treated with a long-acting OCT at 20 mg, a 40 mg OCT, or LAN showed comparable efficacy rates of 13.8%, 15.1%, and 14.6%, respectively. Biochemical response markers remained stable for the duration of the extension study, and mGH and IGF-1 levels were stable from week 36 to week 72 [34].

### Combination therapy

Another study evaluated the efficacy and safety of PAS-LAR in monotherapy or combination therapy with PEGV in patients previously well controlled with first-generation SRLs in combination with PEGV (the PAPE study). This study assessed 61 patients with normal IGF-1 levels (≤ 1.2 × ULN) on first-generation SRLs and PEGV. Initially, the PEGV dose was decreased by 50%. If IGF-1 remained ≤ 1.2 × ULN at week 12, the patients were switched to 60 mg PAS-LAR in monotherapy. If IGF-1 levels were > 1.2 × ULN, the patients were switched to 60 mg PAS-LAR and continued their treatment with the 50% reduced dose of PEGV. At week 12, 15 patients (24.6%) maintained IGF-1 within the reference range; therefore, they were switched to PAS-LAR at 60 mg once monthly in monotherapy. The other 46 patients (75.4%), whose IGF-1 had increased, were continued on the reduced dose of PEGV but in combination with PAS LAR at 60 mg once monthly. At week 24 from the baseline visit, IGF-1 levels decreased to normal in 45 patients (73.8%) (mean IGF-1 of 1.04 × ULN; 95% confidence interval [CI] 0.91–1.17). Normal IGF-1 levels were observed in 14 of the 15 patients (93.3%) receiving PAS in monotherapy and in 31 of the 46 patients (67.4%) receiving PAS-LAR combined with PEGV. The study showed that PEGV dose reduction by 66.1% was possible after 12 weeks of PAS-LAR treatment. Moreover, 67.8% of patients could discontinue their PEGV treatment after 24 weeks. This study indicated an important PEGV-sparing effect, to the extent of complete PEGV discontinuation [44]. A 48-week extension of that study evaluated 53 patients. At weeks 24 and 48, normalized IGF-1 levels were detected in 93.3% of PAS-LAR monotherapy patients. In 10 of them (66.7%) the PAS-LAR dose could be reduced, initially to 40 mg and subsequently—in five patients—to 20 mg. None of the patients required PEGV treatment re-initiation. In the combination therapy group (PAS-LAR and PEGV) IGF-1 normalization rates increased from 67.4% at week 24 to 71.8% at week 48. However, this had required a PEGV dose increase from 47 mg/week to 64 mg/week. Therefore, the cumulative PEGV dose reduction was 52% by week 48, and PEGV had been successfully discontinued in 50.8% of patients, which demonstrates a high efficacy of PAS-LAR [45].

### Retrospective ‘real-life’ studies

PAS-LAR effectiveness was demonstrated in retrospective real-life studies. One of those was an Israeli multicenter study [46] evaluating 35 patients with active acromegaly inadequately controlled with first-generation SRLs, either in monotherapy (*n* = 18) or in combination with PEGV (*n* = 9) and/or cabergoline (CAB) (*n* = 5). One patient received PEGV monotherapy, one patient received PEGV in combination with CAB, and four patients received an SRL in combination with CAB. Two patients had not been on any medical treatment when their PAS-LAR treatment was started; however, they had a history of resistance to first-generation SRLs. All but five of the evaluated patients had undergone neurosurgery, and six had received radiotherapy. At PAS treatment initiation, 30 patients had uncontrolled acromegaly. Five patients had normal IGF-1 levels, but four of them reported persistent headaches. IGF-1 normalization was achieved in 19 patients (54%), partial IGF-1 control (1.0–1.2 × ULN) was achieved in five, and two patients showed substantial IGF-1 reduction (by ≥ 50%, with the levels still > 1.2 × ULN). Overall, 26 patients (74%) benefited from PAS-LAR treatment. Prior to PAS-LAR treatment initiation, six patients had reported persistent headaches, which later completely resolved in four of them and considerably subsided in severity in the remaining two. Six patients achieved a very rapid (after 1–2 injections) and dramatic response to treatment in the form of IGF-1 reduction below the lower limit of normal, which persisted in five of them despite a dose reduction to 20 mg/28 days. A similar excessive response to PAS treatment has been reported in eight drug-naive patients [26] and in patients previously treated with OCT. Lasolle et al. analyzed 15 patients partially resistant to a first-generation SRL in combination with CAB (3.5 mg/week; *n* = 4) or PEGV (100 mg/week; *n* = 11). The existing treatment was replaced with PAS-LAR at 40 mg/month in eight patients and at 60 mg/month in seven patients. At the first follow-up visit (after an average period of 3 months, after 2–6 PAS-LAR injections) treatment efficacy was similar to that achieved with combination therapy (the mean IGF-1 levels were comparable at 1.0 × ULN and 1.1 × ULN, respectively). Eleven out of the 15 patients had IGF-1 < 1.3 × ULN both on combination therapy and on PAS-LAR monotherapy; however, there was a high variability and inter-individual variation in terms of treatment response. At the end of the study, after a mean of 29 months (range 17–34 months), eight patients continued PAS-LAR treatment, with three of the eight meeting the criteria for complete disease control (IGF-1 ≤ ULN and GH ≤ 1 μg/L) [47]. PAS-LAR efficacy was also demonstrated in a study by Witek et al. conducted in 39 patients who had failed to achieve adequate control on a maximum-dose SRL regimen. After six months of PAS-LAR treatment, 24 patients (61.5%) were on a 60 mg dose, and the remaining 15 patients (38.5%) were on a 40 mg dose. IGF-1 level normalization or GH < 2.5 μg/L was achieved in 23 patients (59.0%) and 21 patients (53.8%), respectively, with 16 patients (41%) meeting both of these criteria. Adopting a stricter criterion of biochemical control (GH < 1.0 μg/L), which is widely used for postoperative follow-up of acromegaly patients, decreased the rates of GH normalization to 20.5% (eight patients). Notably, the patients who achieved biochemical control were found to be older than those with inadequate disease control, with no such differences observed between the sexes [48]. Data mentioned above are summarized in Table 1.

**Table 1:** The efficacy of PAS treatment in acromegaly

**Authors**	**Phase/study**	**No of patients**	**PAS LAR dose**	**Duration** **of** **treatment**	**Criteria for the disease control**	**Biochemical response**	**Tumor shrinkage (% volume)**
Colao et al. (2014) [26]	III (core study), CS2305	176	40–60 mg	12 months	GH < 2.5 μg/L and normal IGF-1	31.3%	(> 20%) 80.8%
Bronstein et al. (2016) [39]	III (extension study), CS2305	81	40–60 mg	12 months	GH < 2.5 μg/L and normal IGF-1	17.3%	(> 20%) 54.3%
Gadelha et al. (2014) [42]	III (core study), PAOLA, C2402	130	40 mg	6 months	GH < 2.5 μg/L and normal IGF-1	15%	(> 25%) 18%
			60 mg			20%	11%
Colao et al. (2020) [43]	III (extension study), PAOLA, C2402	111 62	40–60 mg	304 weeks (5.8 years) 268 weeks (5.2 years)	GH < 1.0 µg/L and normal IGF-1	37%	
Gadelha et al. (2019) [34]	III (core study) (extension study)	123 88	10–60 mg	36 weeks 72 weeks	GH < 1.0 µg/L and normal IGF-1	14.6% 14.6%	
Muhammad et al. (2018) [44]	PAPE (core study)	61	60 mg 60 mg + 50% of PEGV dose	24 weeks	IGF-1 ≤ 1.2 × ULN	93.3% 67.4%	
Muhammad et al. (2018) [45]	PAPE (extension study)	53	20–60 mg 60 mg + PEGV	48 weeks	IGF-1 ≤ 1.2 × ULN	93.3% 71.8%	
Shimon et al. (2018) [46]	“real life”	35	20–60 mg	12 months (13.1 ± 5.3)	Normal IGF-1	54%	
Lasolle et al. (2019) [47]	“real life”	15	40–60 mg	29 months (17–34 months)	GH < 1.0 µg/L and normal IGF-1	20%	
Witek et al. (2021) [48]	“real life”	39	40–60 mg	6 months	GH < 2.5 μg/L and normal IGF-1	41%	

### Pasireotide LAR response prediction

The PAPE study additionally evaluated T2-weighted MRI sequences of 47 of its participants. Increased signal intensity was shown in 14 patients (30%), in eight of whom the increase was particularly pronounced (> 50%). Signal hyperintensity on T2-weighted sequences may indicate an adenoma with cystic degeneration or cell apoptosis, which would suggest antineoplastic effects of PAS-LAR. Such effects may enhance PAS-LAR treatment effectiveness; however, long-term treatment may also increase the risk of pituitary insufficiency (hypopituitarism). These observations require verification with larger studies conducted in a larger population [49]. Another study showed that signal hyperintensity on T2-weighted sequences may be predictive of a better response to PAS-LAR. This conclusion was based on the fact that adenomas with high-intensity T2-weighted images showed a better hormonal response over three months of PAS treatment (mean [SD] × ULN were 0.80 [0.60] vs. 0.45 [0.39], *p* = 0.016), despite a lack of tumor size reduction. PAS-LAR-induced cystic degeneration and/or tumor cell apoptosis may diminish disease activity demonstrated by a drop in IGF-1 levels, with relatively large adenomas. On the other hand, patients with a poor response to first- and second-generation SRLs, lower SSTR2 expression, and thus higher IGF-1 levels on PAS-LAR therapy are more likely to achieve tumor size reduction during PAS-LAR treatment [50]. Studies by Iacovazzo et al. demonstrated that patients with no or low SSTR2 expression show resistance to first-generation SRLs treatment, with no such correlation observed with PAS. Conversely, a lack of or low SSTR5 expression was associated with a poor response to PAS. Tumors with low AIP expression were resistant to first-generation SRLs and showed a low SSTR2 expression, whereas no difference was seen in SSTR5 expression. PAS-LAR responsiveness was independent of the level of AIP expression. Moreover, the study showed that in comparison with densely granulated adenomas, sparsely granulated adenomas show a better response to PAS-LAR treatment (67% vs. 80%, *p* = 0.04) [51]. Based on the results of earlier studies, Puig-Domingo et al. proposed an algorithm for medical treatment selection in patients with acromegaly. Those authors recommend a rapid OCT/PAS test with GH assessment at baseline and 2 h after subcutaneous administration of 100 μg OCT or PAS. With a good response to both tested treatments, treatment selection may be further determined by T2-weighted signal intensity. With low-intensity T2-weighted images and a good response in the short OCT test, the treatment of choice may be a first-generation SRL. High-intensity T2-weighted images and a good response in the short PAS test should be an indication to treatment with PAS. Conversely, a poor test response suggests that treatment with PEGV in monotherapy or in combination with PAS-LAR should be considered. Postoperatively, treatment response can be predicted based on the results of histopathology examinations and molecular analyses. In the case of densely granulated adenomas with high expressions of E-cadherin and SSTR2 and low expressions of SSTR5 and Ki-67, the treatment of choice should be first-generation SRLs. In the case of tumors with opposite characteristics, i.e. sparsely granulated adenomas with low SSTR2 and E-cadherin expression, high SSTR5 expression, and low AIP and high Ki-67 expression, the recommended treatment of choice is PAS-LAR (Table 2). However, these recommendations should be verified in larger populations of patients with acromegaly [52]. Chiloiro et al. evaluated the efficacy of PEGV and PAS-LAR in 74 patients resistant to first-generation SRLs therapy. Out of the 41 patients treated with PEGV, disease control was achieved in 35 (85.4%), whereas out of the 33 patients receiving PAS-LAR, disease control was achieved in 23 (69.7%). Large tumors were associated with a worse response to treatment, with IGF-1 levels > 2.3 × ULN (*p* = 0.049), with low or absent SSTR5 expression (*p* = 0.023), and the GH receptor isoform with exon 3 deletion (d3-GHR*)* (*p* = 0.005) [53].

**Table 2:** Radiological, molecular and pathologic factors influencing response to the different SRLs in the medical treatment of acromegaly [50–52]

**Factor studied**	**OCT/LAN**	**PAS**
T2 MRI signal intensity	hypointensive	hyperintensive
granulation pattern	dense	sparse
SSTR2 density	high	low
SSTR5 density	low	high
Ki67 expression	low	high
AIP expression	high	low
E-cadherin expression	high	low

### Pasireotide in acromegaly – adverse effects

The most common side-effects of PAS treatment include hyperglycemia, diarrhea, diabetes, and cholelithiasis [42, 43]. These side effects are similar to those of first-generation SRLs; however, PAS treatment is associated with significantly higher rates of impaired carbohydrate metabolism [26, 42]. Like first-generation SRLs, PAS strongly binds with SSTR2; however, it additionally shows a high affinity to SSTR5 [8]. This characteristic improves the efficacy of PAS with respect to first-generation SRLs; at the same time, it is responsible for carbohydrate metabolism disturbances [26]. Phase III clinical studies in acromegaly patients showed them developing hyperglycemia, often within the first three months of PAS treatment. The hyperglycemia was mostly mild or moderate, often did not require any treatment or was easily controlled with metformin, incretin drugs, other oral antidiabetic drugs, or insulin, and rarely led to treatment discontinuation [26, 38, 42, 43].

Acromegaly-related hypersecretion of GH and IGF-1 induces insulin resistance in peripheral tissues [54]. The improved biochemical control of acromegaly during PAS treatment probably increases insulin sensitivity of tissues, which improves glucose tolerance even with lowered insulin secretion [55]. The risk factors for the development or exacerbation of hyperglycemia during PAS treatment include the presence and severity of carbohydrate metabolism disturbances, elevated glycated hemoglobin (HbA1c) levels, body mass index (BMI) of ≥ 30 kg/m^2^, dyslipidemia, and hypertension prior to treatment initiation, as well as patient age at the start of treatment (≥ 30 years for drug-naive patients and ≥ 40 years for patients treated with first-generation SRLs before PAS treatment initiation) [56]. In a phase II randomized crossover study evaluating the efficacy and safety of subcutaneous PAS in acromegaly, out of the 38 patients with fasting normoglycemia at baseline, 18.4% had abnormal fasting plasma glucose levels and 7.9% had diabetes by the end of the study. Out of the patients who had abnormal fasting plasma glucose levels at the first visit, 11.8% had normal fasting plasma glucose and 52.9% had fasting plasma glucose levels of over 7 mmol/L (126 mg/dL) at the end of the study. Approximately one-fifth (20.3%) of all the patients taking part in the study whose baseline plasma glucose levels were under 7 mmol/L, fasting glucose levels exceeded 7 mmol/L at the end of the study. Two patients (3.3%) were withdrawn from the study due to worsened plasma glucose level control and elevated HbA1c levels [38]. Colao et al. who made a head-to-head comparison of PAS-LAR and OCT-LAR efficacy in drug-naive patients with acromegaly, reported hyperglycemia-related adverse events in 57% of patients treated with PAS-LAR. During 12 months of adequate antidiabetic treatment, HbA1c levels increased by 0.87 percentage points in diabetics, by 0.64 percentage points in patients with prediabetes, and by 0.75% in patients with normal glucose tolerance at baseline. Antidiabetic treatment was necessary in 44.4% of the study population. Hyperglycemia-related adverse events were the reason for study treatment discontinuation in 3.4% of patients [26]. In the PAOLA study 67% of patients treated with 40 mg PAS-LAR and 61% of patients treated with 60 mg PAS-LAR once every 28 days developed hyperglycemia; these rates were by 36.4% and 31.0%, respectively, higher than in the first-generation SRL group. The mean fasting plasma glucose and HbA1c levels increased at the beginning of the study, and then remained stable for six months. Nearly 40% of the patients from either the 40 mg or 60 mg PAS-LAR group (38% and 39%, respectively) required the use of antidiabetic drugs, with this proportion approximately 6.5 times higher than that in the first-generation SRL group. PAOLA study results must be interpreted by taking into account the fact that the patients included in that study had uncontrolled acromegaly inadequately treated with high-dose first-generation SRLs [42]. Therefore, the rates of impaired glucose metabolism from before PAS-LAR treatment initiation were already higher in this study group than those in other studies [26, 42]. Moreover, this study population characteristic caused higher rates of carbohydrate metabolism disturbances and higher fasting plasma glucose and HbA1c levels both during PAS treatment and at study completion, with approximately 40% of patients requiring additional antihyperglycemic drugs and approximately 10% of patients withdrawn from the study due to impaired carbohydrate metabolism [42]. In an extension of the PAOLA study 75–100% of patients who were either pre-diabetic or had normal glucose tolerance at baseline and 31.3–55.3% of patients who were diabetic at baseline showed HbA1c levels below 7% at the end of the study, which demonstrates PAS treatment safety, provided adequate antidiabetic treatment is initiated in case of carbohydrate metabolism disturbances [42, 43]. Overall, the core and extension phases of the PAOLA study showed that 66.7% of patients treated with 40 mg PAS-LAR, 59.7% of those treated with 60 mg PAS-LAR, and 66.1% of those whose PAS-LAR treatment was initiated in the extension study crossover group, required antidiabetic drugs [26, 42, 43]. Moreover, the increase in plasma glucose levels was shown to be reversible after PAS treatment discontinuation [43]. In a multicenter open-label study of the safety and tolerability of PAS-LAR in patients with acromegaly (the ACCESS study) the mean fasting plasma glucose and HbA1c levels showed a clinically significant increase from 100 mg/dL and 5.9%, respectively, prior to treatment initiation to nearly 136 mg/dL and 6.8%, respectively, at month 3 of treatment. The mean fasting plasma glucose and HbA1c levels in patients treated for at least 15 months were 123 mg/dL and 6.3%, respectively. Hyperglycemia-related adverse events were reported in 45.5% of subjects, 13.6% of whom were diagnosed with diabetes [57]. A group of patients with acromegaly resistant to long-acting first-generation SRLs who received PAS-LAR for six months showed a significant increase in HbA1c from 5.6% to 6.2%, with 59% of those patients requiring antidiabetic drugs. During six months of treatment, the proportion of diabetics increased from 10.3% to 46.2%, the proportion of patients with impaired glucose metabolism increased from 61.6% to 92.4%, and the proportion of patients with normal glucose tolerance decreased from 38.5% to 7.7% [48]. A small prospective study by Lasolle et al. assessed the efficacy and safety of PAS-LAR in patients with acromegaly partially resistant to first-generation SRLs, who had been treated with OCT-LAR or a slow-release formulation of LAN in combination with CAB or PEGV. The baseline fasting plasma glucose and HbA1c levels of 101 mg/dL and 5.8%, respectively, increased significantly to 117 mg/dL and 6.3%, respectively, by month 3 of treatment [47]. In a phase IV clinical study by Samson et al. nearly half of the patients with acromegaly (49.7%) had no hyperglycemia that required treatment during the course of PAS-LAR treatment. Moreover, the study was the first one to unequivocally and directly demonstrate the efficacy of incretin drugs in treating PAS-induced carbohydrate metabolism disturbances. Interestingly, the study showed no differences in terms of impaired carbohydrate metabolism between the patients with acromegaly treated with PAS-LAR and those with Cushing’s disease treated with PAS-LAR or subcutaneous PAS [28]. This finding is not consistent with those of other studies, where the rates and severity of hyperglycemia in patients with acromegaly treated with PAS-LAR seem to be lower than those in patients with Cushing’s disease treated with subcutaneous PAS [55, 58]. Overall, despite its considerable hyperglycemic effect, PAS has been shown to be a safe treatment [43]. Carbohydrate metabolism disturbances often develop during PAS treatment, typically during the first 4–12 weeks of treatment [26, 42]. Most cases do not require drug therapy, and those that do are relatively easily controlled with antidiabetic agents. Nonetheless, PAS treatment requires a close monitoring of carbohydrate metabolism markers, which should be measured prior to and during treatment. In those patients who develop hyperglycemia that requires treatment, the antidiabetic drugs of choice are incretins and metformin, with other options including other oral antidiabetic drugs and insulin [26, 42]. Most experts recommend the use of metformin in first-line treatment of PAS treatment-emergent hyperglycemia; nonetheless, some experts suggest incretin drugs [59, 60]. The proportion of patients with acromegaly who must discontinue PAS due to new or exacerbated carbohydrate metabolism disturbances is relatively low (up to 4%), particularly with the use of long-acting PAS formulations [26, 42].

### Adverse events other than hyperglycemia

Petersenn et al. assessed treatment with subcutaneous PAS administered twice a day for 28 days at a total daily dose of 200, 400, or 600 μg in patients with acromegaly as part of a phase II study and reported drug-related adverse events in 75% of patients during study treatment. The most common adverse events included nausea (25%), diarrhea (22%), abdominal pain (12%), and bloating (10%) [38]. An extension of the study showed even higher rates of these adverse events, namely diarrhea (46.7%), nausea (33.3%), abdominal pain (20%), and flatulence (20%), with 13.3% of patients developing cholelithiasis [61]. A head-to-head comparison of PAS-LAR and OCT-LAR in drug-naive patients with acromegaly revealed lower rates of adverse events (other than hyperglycemia) in the PAS-LAR group than in the OCT-LAR group [26]. Overall, 39.3% of patients developed mild-to-moderate diarrhea, 25.8% of patients developed cholelithiasis, and 18.5% of patients developed headaches. Severe adverse events were more common in the PAS-LAR group; however, they were predominantly hyperglycemia-related [26]. The PAOLA study evaluated the efficacy of 40 mg and 60 mg PAS-LAR in a group of patients inadequately controlled with first-generation SRLs. Apart from hyperglycemia-related adverse events, the following adverse events were reported in the 40 mg and 60 mg groups: diarrhea, mostly mild or moderate (in 16% and 19% of patients, respectively), cholelithiasis (10% and 13%), headaches (14% and 3%), nasopharyngitis (6% and 11%), abdominal pain (8% and 8%), and nausea (3% and 6%). The rates of most of the adverse events were lower than those reported in earlier studies, which was most likely due to enrolling a patient population already treated with SRLs. An extension of that study showed the following side effects in the 40 mg and 60 mg PAS-LAR groups: cholelithiasis (in 34.9% and 33.9% of patients, respectively), headaches (28.6% and 6.0%), diarrhea (22.2% and 27.4%), and abdominal pain (15.9% and 16.1%). Serious adverse events were reported in 7.9% and 9.7% of patients, respectively, and were mostly hyperglycemia-related [42]. The ACCESS study assessed the safety and tolerability of PAS-LAR in patients with acromegaly who had either received PAS-LAR as part of earlier studies, had been inadequately controlled with first-generation SRLs, or had received no treatment despite uncontrolled disease. Apart from hyperglycemia, the most common adverse events were gastrointestinal, e.g. diarrhea (in 38.6% of patients), nausea (27.3%), abdominal pain (18.2%), and cholelithiasis (18.2%) [57]. Many studies show lower rates of gastrointestinal adverse events (diarrhea, nausea, abdominal pain, bloating) on PAS-LAR treatment in comparison with those on short-acting subcutaneous PAS formulation [58, 61, 62]. PAS is generally well tolerated, with a safety profile similar to that of first-generation SRLs, except for the higher rates of impaired carbohydrate metabolism, which are clinically significant, nonetheless mostly mild to moderate in severity and relatively easily controlled. Close blood sugar level monitoring is recommended during PAS treatment, and if diabetes develops, adequate treatment should be initiated. Due to the PAS mechanism of action, the formulations of choice in those cases are metformin and incretins or other oral antidiabetic drugs and insulin, if necessary. When no adequate glycemic control can be achieved, PAS dose reduction or complete discontinuation should be considered. Long-acting PAS formulations are preferable due to their lower rates and severity of side effects, better effectiveness of treatment, and higher quality of life [61, 62]. An increased risk of cholelithiasis is typical of all SRLs; however, if cholelithiasis develops, it is rarely symptomatic and usually does not require surgical intervention. Nonetheless, conducting regular gallbladder ultrasound examinations and relevant blood chemistry tests is recommended during PAS treatment. Most important adverse affects of PAS observed in acromegaly are shown in Table 3.

**Table 3:** Most important side effects of PAS treatment

**Side effect**	**Author**	**Phase/Study**	**Incidence (%)**	**PAS dose**
Diabetes	Petersenn et al. [38, 61]	II	5.0–16.7	200–900 µg/d sc
	Colao et al. [26]	III (core study CS2305)	19.1	40–60 mg LAR
	Gadelha et al. [42]	III (PAOLA, C2402)	21.0–26.0	
	Colao et al. [43]	III (PAOLA, extension study)	31.7–40.3	
	Witek et al. [48]	„real-life”	46.2	
	Fleseriu et al. [57]	ACCESS	13.6	40 mg LAR
	**range for PAS LAR**		**13.6–46.2**	
Carbohydrate disorders other than diabetes (described as hyperglycemia, increased blood glucose, IFG, IGT)	Petersenn et al. [38, 61]	II	6.7–10.0	200–900 µg/d sc
	Colao et al. [26]	III (core study CS2305)	28.7	40–60 mg LAR
	Gadelha et al. [42]	III (PAOLA, C2402)	31.0–33,0	
	Colao et al. [43]	III (PAOLA, extension study)	39.7–40.3	
	Witek et al. [48]	„real-life”	46.2	
	Fleseriu et al. [57]	ACCESS	22.7	40 mg LAR
	**range for PAS LAR**		**22.7–46.2**	
Diarrhoea	Petersenn et al. [38, 61]	II	21.7–46.7	200–900 µg/d sc
	Colao et al. [26]	III (core study CS2305)	39.3	40–60 mg LAR
	Gadelha et al. [42]	III (PAOLA, C2402)	16.0–19.0	
	Colao et al. [43]	III (PAOLA, extension study)	22.2–27.4	
	Fleseriu et al. [57]	ACCESS	38.6	40 mg LAR
	**range for PAS LAR**		**16.0–38.6**	
Cholelithiasis	Petersenn et al. [61]	II	13.3	200–900 µg/d sc
	Colao et al. [26]	III (core study CS2305)	25.8	40–60 mg LAR
	Gadelha et al. [42]	III (PAOLA, C2402)	10.0–13.0	
	Colao et al. [43]	III (PAOLA, extension study)	33.9–34.9	
	Fleseriu et al. [57]	ACCESS	18.2	40 mg LAR
	**range for PAS LAR**		**10.0–34.9**	
Headache	Colao et al. [26]	III (core study CS2305)	18.5	40–60 mg LAR
	Gadelha et al. [42]	III (PAOLA, C2402)	3.0–14.0	
	Colao et al. [43]	III (PAOLA, extension study)	9.7–28.6	
	**range for PAS LAR**		**3.0–28.6**	
Abdominal pain	Petersenn et al. [38, 61]	II	11.7–20.0	200–900 µg/d sc
	Colao et al. [26]	III (core study CS2305)	18.0	40–60 mg LAR
	Gadelha et al. [42]	III (PAOLA, C2402)	8.0	
	Colao et al. [43]	III (PAOLA, extension study)	15.9–16.1	
	Fleseriu et al. [57]	ACCESS	18.2	40 mg LAR
	**range for PAS LAR**		**8.0–18.2**	
Back pain	Colao et al. [26]	III (core study CS2305)	7.9	40–60 mg LAR
	Colao et al. [43]	III (PAOLA, extension study)	11.3–20.6	
	**range for PAS LAR**		**7.9–20.6**	
Flatulence	Petersenn et al. [38, 61]	II	10.0–20.0	200–900 µg/d sc
Nausea	Petersenn et al. [38, 61]	II	25.0–33.3	200–900 µg/d sc
	Colao et al. [26]	III (core study CS2305)	13.5	40–60 mg LAR
	Gadelha et al. [42]	III (PAOLA, C2402)	3.0–6.0	
	Colao et al. [43]	III (PAOLA, extension study)	11.1–11.3	
	Fleseriu et al. [57]	ACCESS	27.3	40 mg LAR
	**range for PAS LAR**		**3.0–27.3**	
Vomiting	Colao et al. [43]	III (PAOLA, extension study)	1.6–12.7	40–60 mg LAR
	Fleseriu et al. [57]	ACCESS	13.6	40 mg LAR
	**range for PAS LAR**		**1.6–13.6**	
Anemia	Gadelha et al. [42]	III (PAOLA, C2402)	3.0–6.0	40–60 mg LAR
	Colao et al. [43]	III (PAOLA, extension study)	15.9–16.1	
	**range for PAS LAR**		**3.0–16.1**	
Dizziness	Petersenn et al. [38, 61]	II	6.7–16.7	200–900 µg/d sc
	Colao et al. [26]	III (core study CS2305)	9.6	40–60 mg LAR
	Gadelha et al. [42]	III (PAOLA, C2402)	2.0–8.0	
	Colao et al. [43]	III (PAOLA, extension study)	4.8–12.7	
	Fleseriu et al. [57]	ACCESS	11.4	40 mg LAR
	**range for PAS LAR**		**2.0–12.7**	
Hypoglycemia	Petersenn et al. [61]	II	6.7	200–900 µg/d sc
	Gadelha et al. [42]	III (PAOLA, C2402)	3.0–6.0	40–60 mg LAR
	Colao et al. [43]	III (PAOLA, extension study)	11.1–11.3	
	Fleseriu et al. [57]	ACCESS	13.6	40 mg LAR
	**range for PAS LAR**		**3.0–13.6**	
Alopecia	Colao et al. [26]	III (core study CS2305)	18.0	40–60 mg LAR
	Gadelha et al. [42]	III (PAOLA, C2402)	2.0–6.0	
	Colao et al. [43]	III (PAOLA, extension study)	4.8–12.9	
	**range for PAS LAR**		**2.0–18.0**	
Hypertension	Colao et al. [43]	III (PAOLA, extension study)	6.5–11.1	40–60 mg LAR
Hypercholesterolemia	Colao et al. [43]	III (PAOLA, extension study)	7.9–9.7	40–60 mg LAR
Fatigue	Petersenn et al. [61]	II	6.7	200–900 µg/d sc
	Colao et al. [26]	III (core study CS2305)	9.6	40–60 mg LAR
	Fleseriu et al. [57]	ACCESS	13.6	40 mg LAR
	**range for PAS LAR**		**9.6–13.6**	
Arthralgia	Colao et al. [26]	III (core study CS2305)	9.6	40–60 mg LAR
	Colao et al. [43]	III (PAOLA, extension study)	11.1–14.5	
	**range for PAS LAR**		**9.6–14.5**	

*IFG* improper fasting glucose, *IGT* impaired glucose tolerance

## Pasireotide in the treatment of Cushing’s disease

### Efficacy of pasireotide in the treatment of Cushing’s disease

ACTH-secreting pituitary tumors associated with hypercortisolism express SSTRs [63, 64]. However, previous attempts to use OCT, which is characterized by a high affinity to SSTR2 and is highly effective in acromegaly, in Cushing’s disease have proven ineffective or effective only in a handful of cases. In 2010, Hofland et al. [65] demonstrated that SSTR2 become downregulated in hypercortisolemia, whereas SSTR5 remain active despite cortisol excess. The first randomized clinical trial showing PAS efficacy in the treatment of Cushing’s disease was a phase III study whose results were published in 2012 (NCT00434148). That study was conducted in 162 patients, who were included based on their mean urinary free cortisol (mUFC) levels ≥ 1.5 × ULN. Study subjects were randomized to receive PAS at a total daily dose of either 1,200 μg or 1,800 μg administered in two subcutaneous injections, with the option of having the dose increased, if ineffective, to 1,800 μg or 2,400 μg per day, respectively. After six months of treatment mUFC was within normal limits in 22.2% of patients, including 15% of patients from the group initially receiving 1,200 μg/day and 26% of those initially receiving 1,800 μg/day. Moreover, 15.4% of patients showed a reduction in mUFC excretion by over 50%, which was defined as partially controlled hypercortisolism. After 12 months of treatment with subcutaneous PAS, 19.1% of patients maintained mUFC within normal limits, and a further 9.3% of patients achieved partially controlled hypercortisolism. At the same time, the decrease in mUFC in effectively treated patients was relatively rapid and occurred within the first 3 months of treatment. The study drug was the most effective in patients with baseline mUFC below 5 × ULN; conversely, less than 10% of the patients with the highest UFC (> 5 × ULN) achieved mUFC normalization [58]. An extension of this study conducted in 58 patients, half of whom were characterized by complete biochemical control (mUFC < ULN) and 21% had partially controlled hypercortisolism (a decrease in mUFC by over 50%), showed the rates of complete biochemical control (mUFC < ULN) decrease at month 24 from 50% to 34.5%, with the rate of partial biochemical control at 8.6% [66]. In 2017, Petersenn et al. published an analysis of a long-term five-year treatment with PAS in a group of 16 patients, whose treatment began as part of the phase III study mentioned above. Those authors reported that most of the patients effectively treated with subcutaneous PAS for 12 months would also achieve good long-term control of the disease. Complete biochemical control at year 5 was achieved in 11 of the 16 patients (69%), with further two patients (12.5%) showing evidence of a partial response [67]. Apart from UFC excretion, the phase III study analyzed additional aspects of the effect of subcutaneous PAS treatment on clinical signs and symptoms of Cushing’s disease. In their international study, Pivonello et al. [68] demonstrated that the reduction in UFC levels observed during treatment with PAS was associated with a significant improvement of the clinical signs and symptoms of hypercortisolism. It is worth emphasizing that some of the evaluated parameters, such as BMI, body weight, waist circumference, and blood pressure, showed considerable improvement even if complete biochemical control (defined as mUFC normalization) was not achieved. Improvement of such lipid profile parameters as total cholesterol and low-density cholesterol levels was observed primarily in the patients who achieved mUFC normalization. Therefore, we can assume that some of the patients who achieve only a partial response to PAS treatment may benefit from that treatment also in terms of the complications and comorbidities of chronic hypercortisolemia. This may be particularly important in patients receiving suboptimal treatment or those awaiting a delayed effect of radiotherapy or another surgery. Beneficial effects on other cardiovascular risk parameters, such as significant improvement of the visceral adiposity index and visceral adipose tissue dysfunction, were also shown in a recent study by Albani et al. [69]. Another recent multicenter Italian study conducted in ‘real-world’ clinical settings demonstrated that the best effects of treatment can be achieved with subcutaneous PAS in patients with mild or very mild disease. Analysis of this study group of 31 patients revealed that subcutaneous PAS at doses ranging from 600 to 1,800 μg in such a selected group leads to normalized UFC excretion in over 60% of patients [70]. This is consistent with the results of the relevant pivotal study [58]. Subcutaneous PAS administration two times daily is unquestionably a source of considerable discomfort for patients with Cushing’s disease, which is characterized by easy bruising and subcutaneous tissue infections. Therefore, the use of PAS-LAR in clinical practice and its approval by the European Medicines Agency (EMA) and the Food and Drug Administration (FDA) will significantly improve the comfort of patients with this rare debilitating disease. PAS-LAR may be administered in patients with Cushing’s disease in the form of once monthly injections and is manufactured in 10, 20, 30, and 40 mg ampules. The pivotal PAS-LAR study (NCT01374906) was conducted in 150 patients. One important difference in comparison with the pivotal study of subcutaneous PAS was the fact that the patients who were to receive once monthly injections had mUFC ranging from 1.5 to 5 × ULN, which had been shown to be associated with very low efficacy of the drug in patients with a very severe form of the disease. The study population was randomized into two groups receiving either 10 or 30 mg once every 4 weeks, and after four months of a stable-dose period, in cases of no biochemical control, the dose could be increased to 30 or 40 mg, respectively. At month 7, 41.9% of patients from the group initially receiving 10 mg and 40.8% of patients initially receiving 30 mg achieved UFC normalization. However, by month 12, the rates of biochemical control decreased to 35% and 25%, respectively. Also in the case of PAS-LAR, the peak control rate of 52% was achieved at month 7 in the group with mild-to-moderate disease, with mUFC between 1.5 and 2.0 × ULN, and a decrease in UFC occurred primarily during the first 12 weeks of treatment [27].

### The effect of pasireotide on tumor shrinkage in Cushing`s disease

An estimated 80% of cases of Cushing’s disease are caused by pituitary microadenomas, whereas macroadenomas comprise only about 15%–20% of cases. Therefore, assessing the effect of PAS on tumor size is considerably more difficult in Cushing’s disease than in acromegaly, with the latter being more common and caused predominantly by somatotropic macroadenomas, which are much easier to assess for maximum tumor size and tumor volume. Therefore, a small sample size is a noticeably common feature of studies found in literature and assessing the effects of PAS on pituitary tumor size. The small sample size is associated with the fact that most patients have undetectable or unmeasurable pituitary tumors and are excluded from tumor size analysis studies. [27, 58]. A phase III study assessing the efficacy of subcutaneous PAS in Cushing’s disease demonstrated some extent of corticotropic tumor volume reduction achieved in a subgroup of 75 patients with measurable pituitary tumors (46% of the study population). At month 12 of treatment, the mean tumor volume decreased by 9.1% in the group originally randomized to receive 1,200 μg PAS per day (*n* = 14) and by 43.8% in the group randomized to receive 1,800 μg/day (*n* = 18). A 2020 post hoc analysis of a pivotal trial evaluated the effects of subcutaneous PAS on pituitary tumor size in more detail, and the presented results involved the relevant parameters from 53 patients after 6 months of treatment and 32 patients after 12 months of treatment. Importantly, the study group included only 11 patients who had not undergone surgery and only 6 cases of pituitary macroadenoma (three in the group randomized to receive subcutaneous PAS at 1,200 μg/day and three in the group randomized to receive the drug at 1,800 μg/day). The post hoc analysis showed a mean tumor volume reduction by 5.7% at month 6 of treatment and by 28.6% at month 12, with the evaluable group somewhat smaller at month 12. The 1,200 μg/day subgroup had a mean tumor volume increased by 9.3% at month 6 (*n* = 25) and decreased by 9.1% at month 12 (*n* = 14). The 1,800 μg subgroup showed a mean tumor volume reduction by 19% at month 6 (*n* = 28) and by 43.8% at month 12 (*n* = 18). The proportion of patients who achieved a significant tumor size reduction (by over 25%) was evaluated at the same time points, yielding 37.7% of patients with this outcome at month 6 and 56.2% of patients with this outcome at month 12. Importantly, in the case of the lower total daily dose (1,200 μg) tumor shrinkage was observed more often in the case of smaller tumors (≤ 0.2 cm^3^) than larger ones (> 0.2 cm^3^); this relationship was not observed with the 1,800 μg/day dose. Nonetheless, an analysis of corticotropic pituitary macroadenomas, that is those tumors whose volume reduction is particularly desirable, yielded significantly worse results. Namely, all three patients with corticotropic macroadenomas randomized to the 1,200 μg/day group had their tumors increase in size by month 6 of treatment, and out of the patients randomized to the 1,800 μg/day group one showed tumor volume reduction by 25%, another showed practically no change in tumor size (reduction by 1%), and the third showed an approximately 40% increase in tumor volume [27, 58]. In 2015, Simeoli et al. reported their observations in eight patients from one center involved in a phase III study. A detailed analysis of this patient group showed that the rates of tumor volume reduction by over 25% were higher at up to 62.5% at month 6 and up to 100% at month 12 of subcutaneous PAS treatment. Moreover, a treatment extension to 24 months in two patients resulted in tumor volume reduction by 80% in one of them and complete tumor disappearance in the other. The study was somewhat limited by the fact that seven of the evaluated patients had microadenomas and only one had a corticotropic macroadenoma [71]. The effect of PAS on pituitary tumor volume reduction was also assessed in a phase III study on the efficacy and safety of PAS-LAR. Importantly, the proportion of macroadenomas in that study was considerably higher at 33% of the study population. Data analysis was conducted after 12 months of treatment in those patients whose tumors were measurable both at baseline and at month 12. The group with an initial dose of 10 mg/month (*n* = 35) showed a median tumor volume decrease by 17.8%, and the group with an initial dose of 30 mg/month (*n* = 38) by 16.3%. In the case of macroadenomas the median tumor volume decreased by 14.6% and 11.6%, respectively. A significant tumor volume reduction (by at least 20%) was demonstrated in 43% and 47% of patients, respectively, whereas a significant (≥ 20%) increase in tumor volume was shown in 9% and 11% of patients from the respective study groups [27]. Daniel et al. attempted to use subcutaneous PAS followed by LAR in the treatment of invasive corticotropic tumors associated with Nelson’s syndrome. A group of five patients who received subcutaneous PAS at 600–1,200 μg for four weeks and were subsequently switched to a LAR formulation at 40–60 mg once every four weeks showed no corticotropic tumor volume reduction at week 28 of treatment despite having achieved a significant decrease in ACTH secretion. However, due to the small sample size, the relatively short treatment period, and a switch to a different form of the drug during the study, these study results must be interpreted with caution [72]. Data mentioned above are summarized in Table 4. No correlation between the achieved tumor volume reduction and UFC reduction was demonstrated for corticotropic adenomas. Similar observations were also presented by Italian authors, who emphasized that their study group achieved a similar extent of tumor shrinkage in fully controlled, partially controlled, and uncontrolled patients. Also in the case of PAS-LAR there was no correlation between tumor volume reduction and the degree of biochemical control [27, 58, 71]. This apparent discrepancy suggests that the effect of PAS on tumor size and the suppressing effect of the drug on tumor secretory activity are independent and probably occur via different molecular pathways. One suspected role of PAS is associated with inhibiting cell growth, cell proliferation, and the cell cycle. One of the possible mechanisms of tumor shrinkage in response to PAS is the drug’s inhibiting the expression and function of the vascular endothelial growth factor (VEGF), as has been demonstrated by Zatelli et al. [73]. Another interesting concept explaining the effects of PAS in only some of the cases of Cushing’s disease is associated with the dominant somatic mutations identified in the ubiquitin-specific protease 8 gene (*USP8*). According to some reports [74–77] this mutation may be present in up to 30%–60% of corticotropic tumors, particularly small tumors of a potent secretory activity. The presence of this mutation inhibits epidermal growth factor (EGF) receptor degradation, which consequently enhances EGF signaling. The presence of gene *USP8* mutations is thought to correlate with SSTR5 expression in those microadenomas, and such tumors are thought to be more responsive to PAS treatment. Conversely, the mutation is less common in larger and invasive corticotropic macroadenomas, which are also believed to be less responsive to PAS treatment, with temozolomide possibly more effective in those cases—as has been suggested by some authors—since O6-methylguanine DNA methyltransferase (MGMT) expression is typically considerably less pronounced in those larger, invasive corticotropic tumors [77, 78].

**Table 4:** The effect of PAS treatment on mUFC and tumor volume in Cushing`s disease

**Authors**	**Phase/study**	**No of patients**	**PAS dose**	**Duration of treatment**	**Biochemical response** **(mUFC normalization or ↓mUFC ≥ 50%)**	**Tumor shrinkage** **(% volume)**
Colao et al. (2012) [58]	III (core study) CSOM B2305	162	PAS s.c (1200–2400 µg/day)	12 months	19.1% (mUFC normalization) 9.3% (↓mUFC ≥ 50%)	–9.1% and –43.8%^*^
Hofland et al. (2010) [65]	III (extension study CSOM B2305)	58	PAS s.c (600–2400 µg/day)	12 months	50% (mUFC normalization) 21% (↓mUFC ≥ 50%)	
				24 months	34.5% (mUFC normalization) 8.6% (↓mUFC ≥ 50%)	
Petersenn et al. (2017) [67]	III (extension study) CSOM B2305	16	PAS s.c (300–2400 µg/day)	60 months	68.8% (mUFC normalization) 12.5%(↓mUFC ≥ 50%)	–3.5%^a^
Pivonello et al. 2019 [70]	III (extension study) CSOM B2305	32	PAS s.c (600–1800 µg/day)	6 months	67% (mUFC normalization)	
Lacroix et al. 2018 [27]	III (core study) CSOM G2304	150	PAS LAR (10–40 mg/4 weeks)	7 months 12 months	41% (mUFC normalization) 25–35% (mUFC normalization)	–17.8% and –16.3%^b^

*mUFC* mean urinary free cortisol

^*^In the groups initially randomized to 1200 µg/day (*n* = 14) and 1800 µg/day (*n* = 18), respectively

^a^*n* = 6

^b^In the groups initially randomized to 10 mg/4 weeks (*n* = 35) and 30 mg µg/4 weeks (*n* = 38), respectively

### The management of pasireotide-induced hyperglycemia in Cushing`s disease

Carbohydrate metabolism disturbances, including prediabetes and diabetes, are typical complications of chronic hypercortisolemia and often develop before PAS treatment is initiated. Impaired carbohydrate metabolism is also more commonly observed in Cushing’s disease than in acromegaly [79, 80]. A recent study on the optimal management of PAS-induced hyperglycemia showed the baseline levels of both fasting plasma glucose and HbA1c to be considerably higher in patients with Cushing’s disease than in those with acromegaly [28]. The mechanism in which impaired carbohydrate metabolism develops in Cushing’s disease is complex. Chronic hypercortisolemia leads to the induction of key enzymes of gluconeogenesis, which increases glucose synthesis from non-carbohydrate sources. At the same time, the effect of insulin in peripheral tissues diminishes, particularly in the liver and skeletal muscles, which increases insulin resistance. There is also the contributory effect of cortisol on the central nervous system, leading to an increase in appetite and inhibition of the hypothalamic satiety center, as well as hyperglycemic hormone potentiation, which is most pronounced with respect to glucagon and catecholamines. Chronic hypercortisolemia also disrupts insulin secretion from pancreatic islets, which also contributes to hyperglycemia. The estimated prevalence of diabetes in patients with Cushing’s disease is 40–50%, with further 20–30% of patients meeting the diagnostic criteria for prediabetes, usually in the form of impaired glucose tolerance (IGT) [79, 80]. Diabetes complicating Cushing’s disease has been classified by the American Diabetes Association (ADA) as a “specific type of diabetes due to other causes” [81]. In the pivotal study on subcutaneous PAS, 73% of patients developed hyperglycemia- or diabetes-related adverse events, with severe adverse events (grade 3 or 4 according to the National Cancer Institute [NCI] Common Terminology Criteria for Adverse Events [CTCAE]) reported in 20% of patients. Fasting plasma glucose and HbA1c levels increased soon after the beginning of treatment and then stabilized following antidiabetic treatment initiation. The mean baseline HbA1c levels were 5.8%; at month 6, they have increased to 7.2% in the 1,200 μg/day group and to 7.4% in the 1,800 μg/day group. No further increase in HbA1c levels was observed at month 12 of PAS treatment (7.3% and 7.2%, respectively). It is important to mention that 45.5% of patients on PAS received a new antidiabetic drug, and 41% of patients who had not been on any antidiabetic treatment received their first antidiabetic drug. The rates of hyperglycemia-related adverse events for PAS-LAR were similar to those for subcutaneous PAS, and were 69% overall and 22% for NCI CTCAE grade 3 and 4 adverse events [27, 58]. PAS-induced hyperglycemia was the most common adverse event observed in this study; however, it caused treatment discontinuation in only 5% of patients [27]. Demonstrating that incretin effect inhibition is one of the fundamental mechanisms behind PAS-induced hyperglycemia was the primary purpose for the use of GLP-1 receptor agonists and dipeptidyl peptidase 4 (DPP-4) inhibitors in PAS-induced hyperglycemia treatment [59]. A recent (2021) study by Samson et al. demonstrated that all but 25% of patients with Cushing’s disease who received subcutaneous PAS developed hyperglycemia requiring antidiabetic treatment. It is consistent with clinical observations indicating that carbohydrate metabolism impairment is more common and severe in Cushing’s disease than in acromegaly [28]. The study by Samson et al. also showed that the patients randomized to receive incretin drugs had lower HbA1c levels and fewer hypoglycemic episodes in comparison with those reported in insulin-treated patients. However, it is important to mention that the randomized group of patients with Cushing’s disease in that study was relatively small. Another limitation was the fact that the study evaluated the effects of a short-acting PAS formulation, whereas it is PAS-LAR that is currently used in the treatment of Cushing’s disease. The study directly and prospectively demonstrated the efficacy of incretin drugs in PAS-induced hyperglycemia in patients with Cushing’s disease. Nonetheless, there is still a need for reliable data on the optimal incretin drug, since liraglutide was used as a GLP-1 analog in the study mentioned above. The drugs currently available on the market include once-weekly formulations, such as semaglutide or dulaglutide. Thus, the use of PAS-LAR administered once a month in combination with a GLP-1 receptor agonist administered once a week may improve the comfort of treatment for the patients [27, 28, 82]. The role of DPP-4 inhibitors in the treatment of PAS-induced hyperglycemia in patients with Cushing’s disease is not fully understood. The 2014 guidelines place DPP-4 inhibitors between metformin and GLP-1 receptor agonists, and the treatments in the Samson et al. study mentioned above were initiated in a similar order. However, the fact that SSTR5-mediated GLP-1 inhibition by PAS is the main mechanism behind PAS-induced hyperglycemia suggests that a GLP-1 receptor agonist may be a better candidate for the treatment of choice. This is due to the fact that DPP-4 inhibitors have a generally less potent hypoglycemic effect and can only potentiate the effect of endogenous GLP-1, whose secretion is strongly inhibited by PAS. Therefore, based on the few available studies, DPP-4 inhibitors seem to be possible treatments for hyperglycemia associated with Cushing’s disease alone, since their effect against PAS-induced hyperglycemia is decidedly less pronounced [28, 59].

### Other side effects of pasireotide in Cushing’s disease

Apart from hyperglycemia, the most common adverse event observed during PAS therapy in patients with Cushing’s disease was diarrhea, reported in 58% of patients in the pivotal study for subcutaneous PAS and in 39% of patients receiving PAS-LAR. The rates of *de novo* cholelithiasis were similar for both PAS formulations at 30% and 33%, respectively, and dose-related. Other, less common adverse events included a mild transient elevation in serum aminotransferase levels affecting up to 30% of patients, as well as headaches, abdominal pain, fatigue, and respiratory tract infections. During subcutaneous PAS treatment, clinical manifestations of adrenocortical insufficiency were observed in 8% of patients, and 2% of patients developed a prolonged QT interval (which required no intervention or treatment discontinuation). Moreover, a study evaluating PAS-LAR treatment also showed a decrease in IGF-1 levels; however, IGF-1 remained still within normal limits during the first month of treatment and stabilized by month 7, with no clinical symptoms [27, 58, 83].

### Areas for further study and the prospects of combination therapy

None of the drugs used so far, irrespective of its mechanism of action, has ensured complete biochemical control of hypercortisolemia in every patient with Cushing’s disease. Although the prospect of a combination therapy with the use of two or even three different formulations of various mechanisms of action is very appealing, there are still not enough studies on the efficacy and safety of combination therapies. Moreover, it has not been established whether it would be more beneficial to use two pituitary-directed formulations or a pituitary-directed drug in combination with an adrenal steroidogenesis inhibitor. In both cases, one component of such treatment could be PAS, with its currently optimal administration via once-monthly injections; nonetheless, the data available in the literature are scarce [82, 84]. In 2010, Feelders et al. assessed a group of 17 patients who underwent an 80-day stepwise medical therapy, which began with subcutaneous PAS in monotherapy administered three times daily in incremental doses up to a maximum dose of 750 μg/day. If UFC normalization was not achieved, four weeks later, the treatment regimen was augmented with CAB administered once every two days at an incremental dose up to 1.5 mg every other day. Subsequently, if UFC excretion was still not normalized, 200 mg ketoconazole three times daily was added to the treatment regimen. This combination therapy used three different molecular targets, with PAS acting on SSTR5 in the pituitary, CAB acting on dopamine 2 (D2) receptors, which are also expressed in corticotropic tumors, and ketoconazole inhibiting adrenal steroidogenesis. PAS in monotherapy—despite the relatively low dose—normalized UFC excretion in 5 out of 17 patients (29%), the addition of CAB helped 9 out of 17 patients (53%) achieve biochemical control, and the addition of ketoconazole at a medium dose increased the rates of control to nearly 90% (in 15 out of 17 patients). This triple combination therapy also helped decrease body weight, reduced waist circumference, and lowered blood pressure. Adverse events included a moderate increase in the relative proportion of glycosylated hemoglobin and a decrease in IGF-1 levels below the lower limit of normal in approximately half of the patients [85]. The year 2017 saw the publication of the initial results of the CAPACITY study, which assessed the efficacy of subcutaneous PAS in monotherapy, with a subsequent combination therapy of PAS and CAB. A group of 66 patients in this study received PAS at an initial dose of 1,200 μg/day for 9 weeks, and then the dose was increased to 1,800 μg/day. If this treatment proved ineffective, CAB was added at 0.5 mg and then at 1 mg per day. After 35 weeks of treatment, UFC normalization was observed in 25 of the 66 patients (38%), with 13 patients (20%) having achieved UFC normalization on PAS monotherapy, and further 12 patients (18%) showing UFC normalization after CAB had been added. A total of 70% patients developed hyperglycemia- or decompensated diabetes-related adverse events, and 25% of patients developed cholelithiasis [86]. When it comes to combination therapy, it is important to emphasize that there have been few reports on its use so far, and further studies are necessary to establish the optimal way of combining PAS with CAB, adrenal steroidogenesis inhibitors, or possibly mifepristone. Assessing the efficacy and safety of combining PAS-LAR administered in once-monthly injections with novel steroidogenesis inhibitors (also never before evaluated as part of a combination therapy), such as osilodrostat or levoketoconazole, would be particularly beneficial; as well as establishing optimal doses of these drugs to minimize the risk of excessive aminotransferase activity or QT interval prolongation. One still unexplored, but potentially important, area of study is the issue of potentially reducing the PAS-induced impairment of carbohydrate metabolism by adding osilodrostat or levoketoconazole; however, this requires further studies.

## Other uses of pasireotide

### Neoplasms

Increased SST receptor expression has been shown in a number of solid tumors, and first-generation SRLs were shown to be effective in advanced midgut neuroendocrine tumors [87, 88]. A phase III randomized double-blind study showed a similar efficacy of PAS-LAR and OCT-LAR in controlling refractory carcinoid symptoms; however, progression-free survival was much longer with PAS-LAR treatment [89]. Subcutaneous PAS proved effective in controlling carcinoid symptoms in 27% of patients with advanced neuroendocrine tumors and carcinoid syndrome inadequately controlled with OCT-LAR, who had been included into a phase III open-label study [90]. There have been several case reports of an effective off-label PAS treatment of hypoglycemia in patients with insulinoma [91–94], nesidioblastosis [95], and non-islet cell tumors [92]. To date, PAS efficacy has been demonstrated in some patients with medullary thyroid cancer [96], and a limited clinical benefit of PAS was shown in the treatment of advanced hepatocellular carcinoma [97]. Prostate cancer, colorectal cancer, pancreatic cancer, bladder cancer, and breast cancer have been reported to have a limited expression of SSTR1–4 and an increased expression of SSTR5, which may suggest potential benefits from the use of PAS in these diseases. [7, 98–100]. A study on the use of PAS in malignant melanoma (NCT01652547) has been recently completed; however, its results have not yet been published.

### Hyperinsulinemic hypoglycemia

There have also been reports of effective PAS treatment in patients with postprandial hypoglycemia (dumping syndrome) following bariatric surgery (Roux-en-Y gastric bypass) [101], familial hyperinsulinemic hypoglycemia [102], and diazoxide-resistant hyperinsulinism (congenital isolated hyperinsulinism) [103].

### Postoperative pancreatic fistula prevention

A meta-analysis of five studies demonstrated that PAS did not lower the risk of postoperative pancreatic fistulae following pancreatectomy, but reduced the rates of pancreatoduodenectomy and rehospitalization [104].

### Other pituitary tumors

There have been reports of effective PAS treatment in several cases of Nelson’s syndrome [105] and treatment-refractory prolactinomas [106].

### Polycystic kidney disease

PAS-LAR was shown to be effective in slowing the progression of total liver volume and total kidney volume, without any reduction in glomerular filtration rate, in patients with polycystic kidney disease [107].

Importantly, the uses of PAS listed above, which are referred to as ‘other’ uses, are not based on robust data and cannot be used as grounds for drawing definite conclusions. In some areas, such as postoperative pancreatic fistula prevention, study results are inconclusive. Therefore, prospective randomized studies for the above indications are necessary.
